# The Experience of Various Kinds of Treatment in Pneumonia

**Published:** 1855-09

**Authors:** 


The Experience of Various Kinds of Treatment in Pneumonia.—The author, after recapitulating the leading points of his former paper, considered,

1st. The question as to the influence of blood-letting in the treatment of pneumonia in regard to mortality. He denied that the normal mortality from that disease could be accurately given, showing, from a table he had collected, that it varied from 3 up to 31 per cent. out of some 7000 cases. He particularly alluded to age, sex, and complication as affecting mortality. At the extremes of life it was very fatal, but benignant at intermediate periods. It was more fatal among females; and complications of other diseases, chiefly phthisis and Bright's disease, greatly increased it. Thus a selection of favorable ages only, a diminution of the number of females, in the number of complicated cases, would generally diminish the mortality.

2d. The author then considered the treatment by blood-letting singly, instancing first two series of cases from Bouillaud, which he showed were not fairly selected according to age, sex, and complication. Also, two series of cases from Grisolle, in one-third of which only had blood-letting succeeded in curing the disease; in the remaining, it had failed, necessitating the conjunction of antimonials; lastly, he alluded to cases similarly treated by Dietel, of Vienna; the mean mortality from the blood-letting treatment was 16·5 per cent. Dr. Routh then considered the treatment by blood-letting, combined with tartar emetic, instancing the cases recorded by Dr. Hughes, of Guy's Hospital, and others occurring in the practice of Drs. Walsh, Peacock, and Taylor. These cases appeared to be in no way selected; indeed, as a rule, very unfavorable, the complicated cases amounting, in those of Dr. Hughes, to 51 per cent.; in the others, 53 per cent. He also alluded to some cases similarly treated by Grisolle. The mortality obtained by these gentlemen was—

| | Simple Pneumonia. Per Cent. | All Cases. Per Cent. |
|---|---|---|
| Dr. Hughes | 2·2 | 24 |
| Drs. Taylor, Peacock, and Walsh | 3·2 | 30 |
| Grisolle | — | 25·4 |
| Mean | — | 26 |

---

3d. He then alluded to the treatment by tartar emetic singly, instancing cases from Louis, and Grisolle, and Dietel, giving a mortality, out of 170 cases, of 18 per cent. These cases were remarkable as generally recovering with very little loss of strength; and in comparing this kind of treatment with that of tartar emetic and blood-letting, conjoined by blood-letting singly, the result proved that that by blood-letting and tartar emetic conjoined was the most fatal, because the most depressing.

4th. Dr. Routh then dwelt on the treatment by chloroform, selecting Varentrapp's cases as the best recorded; but even these were not fairly selected, because containing too small a number of females. The mortality he obtained was 4 per cent., or, including some other cases, which he ought not to have omitted, 11½ per cent. A large number of cases collected by Vacherer, Baumgartner, and Helbeing, (193) gave a mortality only of 4¼ per cent., but he could not speak as to their assertion, not having been able to find the original documents.

5th. The author then spoke of the results obtained by dietetic treatment only. These were of two classes, those obtained by homœopaths, (i. e., in those cases where they had been also diagnosed and investigated by legitimate practitioners), and those obtained by experiments directly made by legitimate practioners themselves. From Jessier's cases the mortality was 14 per cent.; from Dietel's experiments, out of 189 cases so treated, the mortality was 7·4 per cent.; Dr. Todd's treatment was also much less energetic. He discouraged blood-letting and tartar emetic, trusting chiefly to the liquor ammonia acetatis, and giving the patient support.

6th. Dr. Routh then proceeded to speak of the treatment which he recommended. The indications were, first, to diminish the general fever, especially the increased cutaneous and pulmonary respiration. The former was effected by the tincture of the root of the aconitum napellus, on the action of which, in small and poisonous doses, he dwelt at length, and especially in reference to its certainty of action and utility as compared with the ordinary tincture of the Pharmacopœia; the latter indication was effected by oleaceous inunctions, which cooled the skin very rapidly.

The second class of indications was to relieve the local symptoms, which was best effected by the employment of Junot's exhausting apparatus, which did all that blood-letting could do, but saved the patient's blood, and by dry cupping or counter-irritation largely, by turpentine, according to Dr. Todd's plan, or blisters followed by repeated dressings of cotton, so as to deprive the system of a large quantity of fluid ingredient. The last class of indications to be fulfilled was that which had reference to the support of the patient. He objected altogether to the 'diète absolue' of the French, recommending the ordinary middle diet of hospitals, or beef-tea from the first, to obviate tendency to death by depression. He occasionally gave small doses of tartar emetic during the first days of the disease, to promote expectoration, and perhaps an alterative mercurial. Under this treatment, he had been generally very successful in pneumonia.

Dr. Webster remarked that more men died of pneumonia than women, the proportion being 13 of the former to 10 of the latter. It was usually fatal among children; but most fatal after the middle period of life, the greatest mortality occurring in persons between 50 and 60. He referred to the occasional termination of pneumonia in gangrene, and of its rarity in sane as compared with insane patients. Thus, in 3102 dissections of sane persons made in the Civil Hospital at Prague, 55 exhibited gangrene of lungs, or 1 in every 56 cases; whereas in 123 dissections of lunatics, lately published by him in the Psychological Journal, 17 cases exhibited gangrene of lungs, or nearly 1 in every 7 autopsies, which makes the ratio eight times greater among insane than ordinary patients. With respect to treatment, he observed that the experience of French, German, or Italian physicians could not be compared with that of physicians in our own country, as the constitutions of the people were different, and consequently the same kind of treatment not applicable. In this country, though bleeding could not be resorted to to the same extent as formerly, it might be still employed, according to circumstances, either locally or generally, followed by tartar emetic and the repeated application of blisters; mercury afterward, to the extent of slight salivation, was most serviceable.—*Dr. Routh—London Lancet.*

